# The Use of Mechanical Bowel Preparation and Oral Antibiotic Prophylaxis in Elective Colorectal Surgery: A Call for Change in Practice

**DOI:** 10.3390/cancers14235990

**Published:** 2022-12-04

**Authors:** Nikoletta A. Petrou, Christos Kontovounisios

**Affiliations:** 1 Department of Surgery, The Royal Marsden Hospital, London SW3 6JJ, UK; 2 Department of Surgery and Cancer, Imperial College London, London SW7 2BX, UK; 3 Department of General Surgery, Chelsea and Westminster Hospital, London SW10 9NH, UK

**Keywords:** colorectal surgery, mechanical bowel preparation, oral antibiotic prophylaxis, surgical site infections

## Abstract

**Simple Summary:**

Patients undergoing elective colorectal surgery are at a higher risk of surgical site infections (SSIs), which delay their recovery, lead to increased morbidity, and result in significant financial burden to the healthcare services. The World Health Organisation (WHO) recommends the use of mechanical bowel preparation (MBP) and oral antibiotic prophylaxis (OAP) to reduce the rates of SSIs. However, there remains international contention on this topic, and the National Institute of Clinical Excellence (NICE) currently recommends against the routine use of MBP and does not address the issue of OAP. We reviewed the current guidelines and most up-to-date evidence from randomised control trials (RCTs) and meta-analyses since the latest NICE guideline update. Recent evidence continues to favour the effectiveness of the combination of MBP and OAP in reducing SSI rates in elective colorectal surgery.

**Abstract:**

Elective colorectal surgery is associated with one of the highest rates of surgical site infections (SSIs), which result in prolonged length of stay, morbidity, and mortality for these patients and have a significant financial burden to healthcare systems. In an effort to reduce the frequency of SSI rates associated with colorectal surgery, the 2018 World Health Organisation (WHO) guidelines recommend the routine use of mechanical bowel preparation (MBP) and oral antibiotic prophylaxis (OAP) in adult patients undergoing elective colorectal surgery. However, this recommendation remains a topic of debate internationally. The National Institute of Clinical Excellence (NICE) guidelines, last revised in 2019, recommend against the routine use of MBP and do not address the issue of OAP. In this communication, we reviewed the current guidelines and examined the most recent evidence from randomised-control trials (RCTs) and meta-analyses on the effect of MBP and OAP on SSI rates since the 2019 NICE guideline review. This recent evidence clearly demonstrated an SSI-risk-reduction benefit with the additional use of OAP and the combination of MBP and OAP in this group of patients, and we therefore highlight the need for change of the current NICE guidelines.

## 1. Introduction

Surgical site infections (SSIs) pose a substantial clinical and financial burden to healthcare systems worldwide. The impact of these healthcare-associated infections on patient length of stay, morbidity, and mortality is significant [1]. Elective colorectal surgery is by its nature a clean–contaminated procedure and is predictably associated with one of the highest SSI rates among elective operations. In the U.K. alone, data published by the U.K. Health Security Agency reviewing the incidence of SSIs across NHS Hospitals in England showed a rise in SSI risk for colorectal surgery from 7.7% to 9.9% in 2020–2021 [2]. 

As SSIs are largely preventable, it becomes essential to reach a consensus on interventions that can be made to reduce their frequency and clinical impact. The World Health Organisation (WHO) guidelines published in 2016 and updated in 2018 recommend the routine use of oral antibiotic prophylaxis (OAP) combined with mechanical bowel preparation (MBP) in adult patients undergoing elective colorectal surgery [3]. However, the combination use of OAP and MBP to reduce surgical site infection is not currently recommended by the National Institute of Clinical Excellence (NICE) [4]. Although parts of the guideline were further revised and included in the 2019 updated publication, no changes were made in the above recommendations. There is, however, increasing evidence from randomised-control trials (RCTs) and metanalyses (MAs) to support the routine use of the combination of MBP and OAP. 

In this review, we aim to examine the recent evidence on the combination use of preoperative MBP and OAP and compare the current recommendations among international societies. 

## 2. Materials and Methods

The guidelines of the National Institute of Clinical Excellence (NICE) [4], World Health Organisation (WHO) [3], American Society of Colon and Rectal Surgeons (ASCRS) [5], Association of Coloproctology of Great Britain & Ireland (ACPGBI) [6], Australian Cancer Council [7], and the Japan Society for Surgical Infection [8] were accessed to seek the most up-to-date statements and recommendations on the topic of preoperative oral antibiotics and mechanical bowel preparation combination in patients undergoing elective colorectal surgery. We also reviewed the most recent published relevant RCTs in the field since the last NICE guideline update of 2019.

Data of interest included year of publication, comments, clinical practice recommendations on the use of preoperative oral antibiotic prophylaxis alone, comments on the use of mechanical bowel preparation alone, and comments on the use of their combination. The guidelines and recommendations were reviewed by the two authors (N.P. and C.K.), who independently identified discrepancies and areas of further research. The authors subsequently made recommendations in areas where the current NICE guidelines could be adjusted to account for the most recent evidence. 

## 3. Results

### 3.1. The 2018 Wold Health Organisation (WHO) Guidelines 

The 2018 WHO global guidelines for the prevention of SSI recommend the routine combination use of MBP and OAP in adult patients undergoing elective colorectal surgery to reduce the risk of SSIs [3]. The guidelines further recommend that MBP alone (without OAP) is not indicated for reducing SSI risk in this group of patients. The WHO guidelines were initially published in 2016 and further reviewed and updated in 2018, and the above recommendations remained unchanged. 

### 3.2. International Guidelines

The 2019 American Society of Colon and Rectal Surgeons (ASCRS) Clinical Practice Guidelines recommend the use of combined MBP and OAP for elective colorectal resections [5]. They do not recommend preoperative MBP alone without OAP and equally do not recommend OAP alone without MBP (based on paucity of high-quality evidence). The American Society for Enhanced Recover (ASER) and the Perioperative Quality Initiative (POQI) joint consensus statement (2017) endorses the routine use of MBP and OAP before elective colorectal surgery [9]. Similarly, the Japan Society for Surgical Infection guidelines (2021) also recommend the combined use of MBP and OAP [8]. 

In contrast, the U.K. and Australia recommend against the use of MBP. In particular, the 2017 guideline of the Association of Coloproctology of Great Britain & Ireland (ACPGBI) recommends that routine use of MBP is to be avoided in elective colorectal surgery [6]. They recommend a single dose of broad-spectrum IV antibiotic prophylaxis prior to commencement of surgery to minimize the risk of SSI. Moreover, the 2018 Australian Cancer Council clinical practice guidelines also do not recommend the routine use of MBP in colonic surgery [7]. 

There is no official consensus statement or guidelines produced by the European Society of Coloproctology (ESCP). However, a 2017 survey that was conducted among 462 European Surgeons noted that 29.6% routinely used MBP prior to colonic surgery and 77% prior to rectal surgery. Oral antibiotics were only routinely used by less than 10% of respondents, whereas perioperative intravenous antibiotics were used by 96%. [10]

### 3.3. The 2008 NICE Guidelines

The 2008 CG74 NICE guideline on the prevention and treatment of SSIs recommended against the use of MBP routinely to reduce the risk of SSIs [4]. In terms of antibiotic prophylaxis, the guideline also recommended considering giving a single intravenous (IV) dose of antibiotic prophylaxis on induction of anaesthesia. The guideline did not comment on the use of OAP or the combination of MBP and OAP. In 2019, the CG74 NICE guideline was updated. The new NG125 guideline, replacing CG74, continued recommending against MBP and advocating for a single IV antibiotic dose on induction of anaesthesia [4]. 

At the time of preparing the 2019 guideline update, NICE also completed an exceptional review on the use of OAP and MBP combination on SSI impact in elective colorectal surgery, assessing the evidence available at the time [11]. Taking into account evidence from systematic reviews and RCTs published between 2014–2019, published evidence provided by stakeholders, the findings of the original 2008 review, and the input of topic experts, the NICE panel concluded that the new evidence indicated that combination of MBP and OAP was the most effective approach to reducing SSI risk in elective colorectal surgery. The panel therefore recommended that the NG125 guideline needed to be updated to consider the new evidence [11]. There have been no further updates on this topic since 2019. 

The current international recommendations on the use of MBP and OAP in elective colorectal surgery are summarised in Table 1. 

## 4. Discussion

The use of MBP and OAP either alone or in combination prior to elective colorectal surgery has been a longstanding topic of debate. As noted from the comparison of the international guidelines, universal consensus is yet to be reached. 

### 4.1. Combination of MBP and OAP Effect in SSI Risk Reduction

MBP alone has not been shown to reduce the risk of SSIs in elective colorectal surgery. Rising evidence from RCTs and meta-analyses, however, supports the routine use of a combination of MBP and OAP. A 2018 meta-analysis by McSorley et al. reviewing 14 RCTs and 8 observational studies, with a total number of 57,207 patients, concluded that OAP in combination with MBP and IV antibiotics was superior to MBP and IV antibiotics alone in reducing SSI in elective colorectal surgery and was associated with reduced frequency of anastomotic leak, ileus, reoperation, readmission, and mortality [12]. 

A further 2018 meta-analysis by Toh et al. that included 8458 patients from RCTs only concluded that MBP combined with OAP was associated with the lowest risk of SSI, compared to MBP alone [13]. Similarly, a 2019 meta-analysis by Rollins et al. of 69,517 patients from RCTs and cohort studies comparing OAP with or without the use of MBP concluded that the combination approach was associated with a significant reduction in SSI compared to MBP alone. There was no significant difference between the combination of MBP and OAP and OAP alone in terms of SSI and anastomotic leak rates; however, there was a significant reduction in 30-day mortality with the combination approach over OAP alone following elective colorectal surgery [14]. 

RCTs published since the last NICE guideline review of 2019 all favour the use of OAP in elective colorectal surgery. A 2019 multi-centre RCT from the Netherlands by Abis et al. compared administration or absence of OAP in addition to the standard IV perioperative prophylaxis in patients that underwent elective colorectal surgery. MBP was given in all patients who had left-sided surgery (left colectomy, sigmoid resection, anterior resection). The findings of the trial showed superiority of the OAP addition in reducing infectious complications after colorectal resection [15]. 

Moreover, a RCT by Uchino et al. (2019) investigating 335 patients with Crohn’s disease who underwent elective bowel resection and received preoperative MBP noted significantly lower incidence of incisional SSI in patients who also received OAP, and their multivariate analysis concluded that absence of OAP was an independent risk factor for incisional SSI [16]. The combination of MBP and OAP versus MBP alone was further compared in a controlled clinical trial by Vadhwana et al. (2020) of 311 patients. They observed a cost-effective and significant reduction in the rate of SSIs and length of stay in the combination group compared to MBP alone [17]. Finally, a 2021 multi-centre RCT of 600 patients by Papp et al. compared MBP with or without OAP prior to elective colorectal surgery and confirmed a statistically significant reduction in the rate of SSI and anastomotic leak in the MBP and OAP combination group [18]. 

### 4.2. OAP’s Role in SSI Risk Reduction

In addition to the preoperative combination of MBP and OAP, the use of OAP alone in elective colorectal surgery has also been extensively evaluated. In a 2020 meta-analysis by Nelson et al. comparing the addition of OAP to IV antibiotics to IV antibiotics alone, they noted that the combined route was superior in reducing SSI rates compared to OAP or IV antibiotics alone [19]. In a large cohort study by Klinger et al. (2019) reviewing the data from the American College of Surgeons National Surgical Quality Improvement Program, a total of 27,804 patients were analysed. Significantly fewer SSIs were seen in patients who received dual preparation with MBP and OAP compared to receiving no preparation at all. MBP alone did not confer a risk benefit in reducing SSI; however, OAP alone did significantly reduce SSI rates compared to no preparation [20].

Recent RCTs also favour the additional use of routine OAP in elective colorectal surgery. A multi-centre RCT by Espin Basany et al. (2020) of 565 patients not receiving preoperative MBP noted that patients who received OAP for 1 day before their elective colorectal surgery, in addition to the IV antibiotics given at induction, had significantly reduced incidence of SSIs compared to patients who received IV antibiotics on induction alone [21]. They therefore recommended the routine administration of OAP on the day before colorectal surgery. More recently, a 2021 single-centre RCT of 116 patients by Rybakov et al. also noted significantly reduced risk of SSI with the use of OAP and IV antibiotics at induction compared to IV antibiotics alone [22].

### 4.3. Choice of Oral Antibiotic Prophylaxis

In practice, there remains no consensus for the optimal regimen of OAP, and a high degree of variation of antibiotic protocols is observed in keeping with the local and regional hospital policies. A 2014 Cochrane review of 43,451 participants within 260 RCTs using prophylactic antibiotics in elective and emergency colorectal surgery by Nelson et al. [23] determined that oral or IV antibiotic choice should include both aerobic and anaerobic cover, as both showed statistically significant reductions in the rate of surgical wound infections

Taking into consideration the evidence examined, we propose that the current NG125 NICE guideline is revised to recommend in favour of the combination MBP and OAP approach in patients who are undergoing elective colorectal surgery. The NG125 NICE guideline should also expand its antibiotic recommendations (currently limited to IV antibiotic prophylaxis only) to account for the SSI-risk-reduction benefit with the additional administration of OAP in this group of patients, as has been demonstrated via the multiple published RCTs.

## 5. Conclusions

The combination use of MBP and OAP in elective colorectal surgery raises multiple considerations that need to be addressed, including the issue of antimicrobial resistance, the appropriate choice of antibiotic and optimal duration of OAP course, and the cost effectiveness of the MBP and OAP combination. Overall, however, current evidence supports the routine use of a MBP and OAP combination prior to elective colorectal surgery. The most recent evidence continues to align with the 2018 recommendations of the World Health Organisation (WHO) in contrast to the NICE guidelines that remain in need of revision. 

## Figures and Tables

**Table 1:** Current recommendations on the use of MBP and OAP in elective colorectal surgery.

Guideline	Year of Publication	Mechanical Bowel Preparation (MBP)	Oral Antibiotic Prophylaxis (OAP)	Perioperative Intravenous (IV) Antibiotics
World Health Organisation (WHO) ^a^	2018	(x)	(x)	(x)
National Institute of Clinical Excellence (NICE) ^b^	2019			(x)
American Society of Colon and Rectal Surgeons (ASCRS) ^c^	2019	(x)	(x)	(x)
Association of Coloproctology of Great Britain & Ireland (ACPGBI) ^d^	2017			(x)
Australian Cancer Council ^e^	2018			(x)
Japan Society for Surgical Infection ^f^	2021	(x)	(x)	(x)

(x) indicates the presence of the recommendation in the guidelines. ^a^ World Health Organization (2018). Global guidelines for the prevention of surgical site infection [3]. ^b^ National Institute for Health and Care Excellence (NICE) (2019). Surgical site infections: prevention and treatment: Recommendations. NG125 [4]. ^c^ Clinical Practice Guidelines Committee of the American Society of Colon and Rectal Surgeons [5]. ^d^ Association of Coloproctology of Great Britain & Ireland (ACPGBI): Guidelines for the Management of Cancer of the Colon, Rectum, and Anus [6]. ^e^ Clinical practice guidelines for the prevention, early detection and management of colorectal cancer. National Health Medical Research Council [7]. ^f^ Committee for Gastroenterological Surgical Site Infection Guidelines, the Japan Society for Surgical Infection [8].

## Data Availability

All reported data have been appropriately referenced, with no original data being reported by the authors.
